# Dynamic Photoresponse of a DNTT Organic Phototransistor

**DOI:** 10.3390/s23052386

**Published:** 2023-02-21

**Authors:** Marcello Campajola, Paolo Di Meo, Francesco Di Capua, Paolo Branchini, Alberto Aloisio

**Affiliations:** 1 Istituto Nazionale di Fisica Nucleare (INFN), Sezione di Napoli, Via Cintia, 80126 Napoli, Italy; 2 Department of Physics “E. Pancini”, University of Naples “Federico II”, Via Cintia, 80126 Napoli, Italy; 3 Istituto Nazionale di Fisica Nucleare (INFN), Sezione di RomaTre, Via della Vasca Navale 84, 00146 Roma, Italy; 4 CNR-SPIN, Via Campi Flegrei 34, 80078 Pozzuoli, Italy; 5 Task Force di Bioelettronica, University of Naples “Federico II”, Via Cintia, 80126 Napoli, Italy

**Keywords:** organic field-effect transistors (OFET), organic phototransistor (OPT), dinaphtho-thieno-thiophene (DNTT), fast photoresponse, low-voltage operations OPT, random telegraph signals (RTS)

## Abstract

The photosensitivity, responsivity, and signal-to-noise ratio of organic phototransistors depend on the timing characteristics of light pulses. However, in the literature, such figures of merit (FoM) are typically extracted in stationary conditions, very often from IV curves taken under constant light exposure. In this work, we studied the most relevant FoM of a DNTT-based organic phototransistor as a function of the timing parameters of light pulses, to assess the device suitability for real-time applications. The dynamic response to light pulse bursts at ~470 nm (close to the DNTT absorption peak) was characterized at different irradiances under various working conditions, such as pulse width and duty cycle. Several bias voltages were explored to allow for a trade-off to be made between operating points. Amplitude distortion in response to light pulse bursts was also addressed.

## 1. Introduction

Organic field-effect transistors (OFETs) are attracting the interest of the scientific community as a valid alternative to standard semiconductor-based devices. This is due to their several interesting properties, such as cost-effective fabrication over large areas, flexibility, and light weight. The recent progress in OFET fabrication techniques has led to the development of devices with high field-effect mobilities *μ*, low threshold voltages V_th_, and high current on/off ratios I_on/off_. OFETs can be successfully employed as building blocks in electronic circuits, as well as sensors in applications such as chemical and biological sensing, gas analysis, and pressure monitoring [1,2,3,4]. Several works also demonstrated how OFETs can be efficiently used as organic phototransistors (OPTs). The potential of such devices lies in the good photosensitive properties of organic semiconductors, combined with the transistor intrinsic amplification capability [5,6]. Fields of application of OPTs also include optical memory, light communication [7,8,9], and radiation detection and dosimetry in radiotherapy [10,11,12]. Several organic semiconductor materials have been investigated for OPT development, including TIPS-pentacene and dinaphtho[2,3-b:2′,3′-f]thieno[3,2-b′]thiophene (DNTT). For those materials, field-effect mobility comparable to that of amorphous silicon, typically *μ*~1 cm^2^/Vs, low turn-on voltage, and on/off current ratio larger than 10^6^ have been reported [13,14,15].

The origin of the photoresponse in OPTs is usually attributed to the increase in the minority carrier trap generation rate under light exposure [16,17]. For a p-type device (i.e., hole-transporting device), the accumulation of photogenerated electrons in trap states in the phototransistor channel causes a shift in the threshold voltage, resulting in multiple holes injected into the channel for a single photogenerated carrier pair. Such a mechanism is known as photoconductive gain. The transverse electric field induced by the gate plays the role of helping to segregate photogenerated electron–hole pairs, decreasing the probability of recombination. The photoconductive gain is recognized as a slow process: phototransistors that exhibit high photoconductive gain usually do not operate as fast as a photodiode with similar carrier transit time [16,18]. Hence, characterizing the dynamic photoresponse to transient light pulses is of paramount importance for devices of interest for real-time applications.

The organic thin-film charge photogeneration has been investigated in the range from picoseconds to seconds after excitation [19,20]. The optical response has been also investigated in depth in OPTs. Among others, we mention here studies on devices based on P3OT [21], PQT-12 [22], PBDFTDTBT [23], P3H3 [24], PDVT-8/PC_61_BM [25], DNTT [26,27,28], and TIPS-pentacene [15]. Most of these works studied the performance of OPTs under quasi-static light conditions at different irradiance, whereas a characterization of the device dynamic response to short light pulses at low irradiance has been barely investigated. Quasi-static characterization is useful for comparing the performance of different materials and devices; however, it is not sufficient for assessing their suitability for detection of short light pulses.

In this paper, we present a systematic characterization of a DNTT-based OPT, specifically developed for real-time radiation detection and dosimetry applications [12,29,30], where fast photoresponse to weak, fast light pulses is expected to be a key feature. Specifically, we report on the response to ~470 nm light pulses, under various timing conditions, at different irradiances and operating points. Several figures of merit (FoM), such as photosensitivity, responsivity, and signal-to-noise ratio, have been investigated. In all the explored regions, these quantities strongly depend on the timing characteristics of the light pulses. In this work, we have characterized them as a function of the pulse width, frequency, and duty cycle, studying the correlation with the bias voltages. We also have studied stress and distortion effects in the photoresponse when light pulse bursts are applied, to evaluate the exploitation of such devices in applications where random light pulse sequences are expected.

## 2. Materials and Methods

### 2.1. Organic Phototransistor Layout

The device under characterization is a thin-film transistor based on DNTT. The device was designed, engineered, and realized at the CNR-IMM laboratory (Rome, Italy). Details on the fabrication process are given in Refs. [29,30]. Here, we only recall some useful information. The OPT was fabricated in a bottom-gate/top-contact configuration on a 100 µm thick substrate of polyethylene-naphthalate. The gate is made by a 70 nm thick Al layer. Source and drain are made by 30 nm thick interdigitated finger electrodes of Au. The semiconductor is 50 nm thick. A dielectric layer, made of a 600 nm thick fluoropolymer-based material (Cytop^TM^), separates the semiconductor from the gate. The whole structure is encapsulated within a 240 nm thick layer of Cytop. 

**Figure 1:**
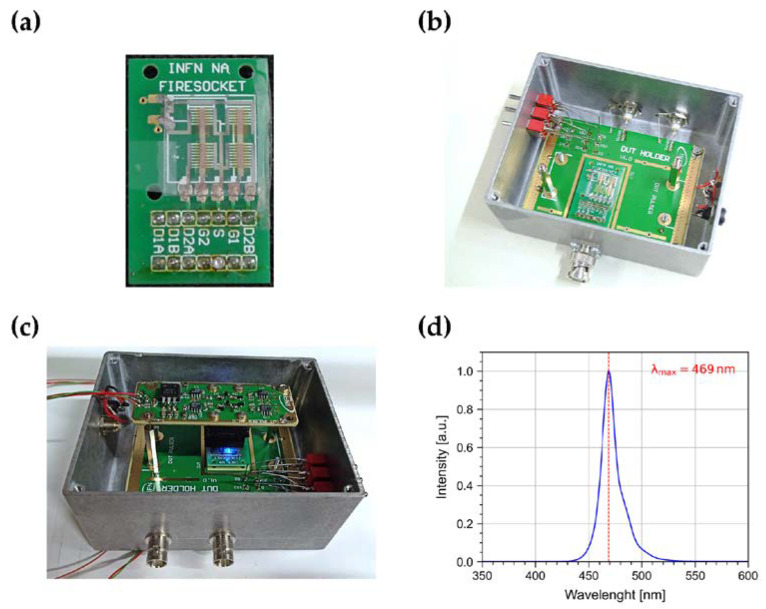
Pictures of (**a**) the 2 × 2 OPT matrix bonded on a custom-made socket; (**b**) the OPT matrix on the socket arranged on a motherboard within an aluminum box used as Faraday cage; (**c**) the aforementioned setup with a LED source and its driver circuit mounted on top of the OPT. (**d**) Emission spectrum of the LED source.

OPT are arranged in a 2 × 2 matrix (see Figure 1a). Each OPT has a channel length L = 100 µm and a width W = 40 mm. The active area A = L × W is 0.04 cm^2^.

### 2.2. Electrical and Photoresponse Characterization Setup

The device was bonded on a custom-made socket (Figure 1a) arranged on a motherboard enclosed in a Faraday cage (Figure 1b), which also acts as a black box to shield the samples from environmental light. 

The electrical characterization was performed by means of a B1500A Semiconductor Device Parameter Analyzer, equipped with three source meter units (SMUs) connected to the device source, gate, and drain. The SMUs were used to bias the OPT and to collect transfer and output curves. 

For the dynamic photoresponse characterization, the device was illuminated with an LED source (Broadcom HLMP-KB45-A0000) installed on the top of the motherboard housing the device under test (DUT) within the Faraday cage (Figure 1c). 

The LED emission spectrum (Figure 1d) was characterized by means of a CCD-based Ocean Insight Spectrometer with a sub-nm resolution. Such a LED was chosen due to its emission wavelength peak (469 nm) close to the absorption peak of the DNTT: ~450 nm (see Ref. [29]). The LED current was controlled by a driver circuit connected to a function generator used to supply control pulses with variable timing. An irradiance in the range from a few nW/cm^2^ to about 10 µW/cm^2^ was achieved with a collimated flat-top spot with a diameter of ~4 mm on the DUT surface. The irradiance was calibrated by means of a Thorlabs PM100USB power meter equipped with a S120C silicon photodiode.

The B1500A unit was used in the “I/V-t sampling measurement” mode [31] to sample at a rate of 20 Hz the OPT drain current trend under illumination. 

All measurements were conducted under ambient atmospheric conditions at room temperature. 

## 3. Results and Discussion

### 3.1. Electrical Characterization in Dark

**Figure 2:**
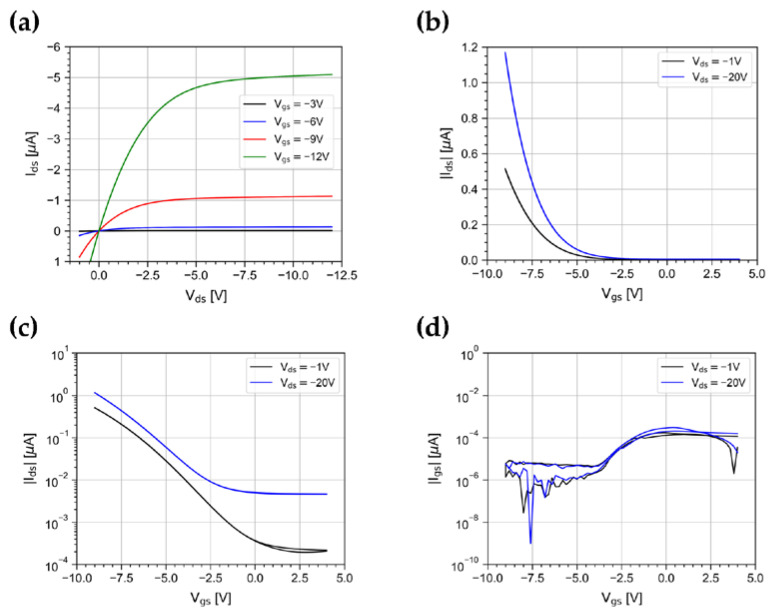
(**a**) Output characteristics for V_gs_ varying between −3 V and −12 V in steps of 3 V, and transfer characteristics in linear (V_ds_ = −1 V) and saturation regimes (V_ds_ = −20 V) represented in (**b**) linear and (**c**) log-y scale. (**d**) Gate leakage current curves for V_ds_ = −1 V and V_ds_ = −20 V.

We measured the output and transfer characteristics of the device in the dark. Output curves (Figure 2a) were acquired with a forward and reverse drain voltage V_ds_ scan in the range [+1 V, −12 V], for gate voltage V_gs_ values between −3 V and −12 V in steps of 3V. Transfer curves (Figure 2b,c) were acquired in linear (V_ds_ = −1 V) and saturation regimes (V_ds_ = −20 V) with a forward and reverse V_gs_ scan in the range [+4 V, −9 V]. 

The curves show a typical p-type field-effect transistor behavior: outputs clearly show the modulation effect due to V_gs_, good linearity for low V_ds_, and saturation characteristics at high V_ds_. Curves show minimal hysteresis between forward and reverse gate-voltage sweeps in both the linear and saturation regimes. The gate leakage current I_gs_ (Figure 2d) is lower than 10^−10^ A, indicating a high-quality gate insulation.

From the curves, we extracted several characteristic parameters of the DUT [32]. The field-effect mobility, computed in the linear regime (V_ds_ = −1 V), is ~ 0.5 cm^2^ V^−1^ s^−1^ at V_gs_ = −12 V. The threshold voltage is around −10 V. The onset voltage is in the range +2 V to −3 V. The subthreshold slope is ~2.4 V dec^−1^. The log(I_on_/I_off_) ratio is around 5, where I_on_ is measured at V_gs_ = −12 V. All the extracted parameters in the explored ranges are in good agreement with values reported in the literature for DNTT-based OFETs [13,14,26,27,28,29,30].

### 3.2. Dynamic Photoresponse

**Figure 3:**
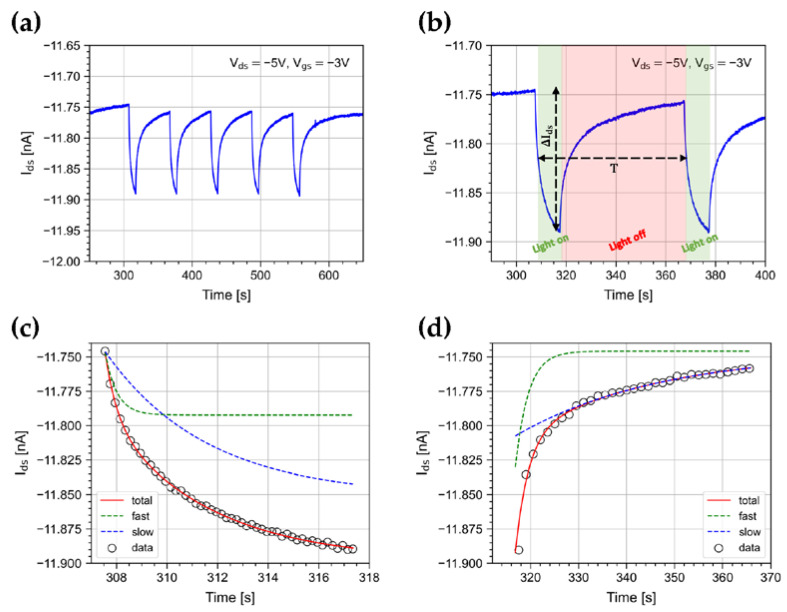
(**a**) I_ds_ current when illuminating the device with a 5-pulse burst with a period of T = 60 s. (**b**) Details of the first two pulses of the burst. Data and best-fit curve with details on the fast and slow exponential function components for the (**c**) signal growth and (**d**) decay.

We measured the device photoresponse to light bursts at ~470 nm. Upon illumination, the device shows a fast switching of the drain current. As an example, we show in Figure 3a the drain current measured when illuminating the device with a burst of 5 pulses at an irradiance of 500 nW/cm^2^, with a repetition period T = 60 s (see Figure 3b) at V_ds_ = −5 V and V_gs_ = −5 V. The signal shows a temporal development that can be parameterized as the sum of two exponentials [33,34,35,36]. The measured characteristic times are $\tau_{fast}=\left(0.461\pm 0.005\right)$ s and $\tau_{slow}=\left(4.02\pm 0.02\right)$ s, as shown in Figure 3c. When the light is turned off, the device response drops very slowly, with characteristic times $\tau_{fast}=\left(2.71\pm 0.03\right)$ s and $\tau_{slow}=\left(30.18\pm 0.15\right)$ s, as shown in Figure 3d. Both rise and fall times are almost constant within the burst. The slow return of I_ds_ to the initial conditions when light is turned off is due to the time it takes for trapped photogenerated traps to recombine [33]. 

We investigated several key FoMs, as well as studied the correlation of the pulse timing and burst structure with the irradiance and the bias voltages, in order to characterize the response in real-time applications.

The photosensitivity P and the photoresponsivity R were computed as follows [1,15]:

(1) $$P=\frac{{\Delta I}_{\mathrm{ds}}}{I_{\mathrm{ds}}^{\mathrm{dark}}\;},$$

(2) $$R=\frac{{\Delta I}_{\mathrm{ds}}}{A\times \mathrm{IRR}},$$

where ${\Delta I}_{\mathrm{ds}}=I_{\mathrm{ds}}^{\mathrm{light}}-I_{\mathrm{ds}}^{\mathrm{dark}}$, and $I_{\mathrm{ds}}^{\mathrm{light}}$ and $I_{\mathrm{ds}}^{\mathrm{dark}}$ are the drain current in dark conditions and under illumination, respectively; A is the active device area; and IRR is the irradiance of the light source. R and P depend on the transistor layout and on the polarization conditions [29].

**Figure 4:**
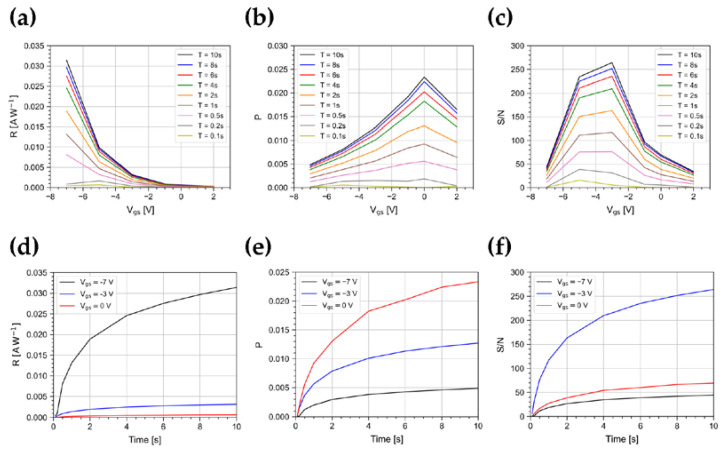
(**a**) Responsivity, (**b**) photosensitivity, and (**c**) signal-to-noise ratio as a function of V_gs_ for V_ds_ = −1 V for light pulse widths from 0.1 s to 10 s. (**d**) Responsivity, (**e**) photosensitivity, and (**f**) signal-to-noise ratio as a function of the light pulse width at V_gs_ = −7, −3, 0 V and V_ds_ = −1 V. All these FoMs are shown at an irradiance of 500 nW/cm^2^.

Figure 4 shows the trend of R and P as a function of V_gs_ for V_ds_ = −1 V and for pulse widths up to 10 s at a constant irradiance of 500 nW/cm^2^. The values shown in the plot are the average taken on bursts of 5 pulses. The responsivity has a minimum as V_gs_ approaches 0 V, while it gradually increases up to 0.03 AW^−1^ at V_gs_ = −7 V, as a result of the increasing exciton dissociation rate due to the transversal electrical field in the channel [27,37]. The maximum value of P is 0.024, reached at V_gs_ = 0 V. This is near the turn on voltage, as a result of the abundance of photogenerated carriers over scarce field-generated carriers. At more negative V_gs_ values, the photoresponse is subdued to the increasing number of field-induced charge carriers [27].

Furthermore, we measured the signal-to-noise ratio S/N, where the noise *N* is evaluated as the RMS of the dark current measured immediately before the arrival of the light pulse. Figure 4c shows the trend of S/N as a function of V_gs_ and for different pulse widths. The maximum value is ~260, reached at V_gs_ = −3 V for a pulse width of 10 s. For a width shorter than 0.5 s, the photoresponse reduces, and the highest S/N ratio occurs at V_gs_ = −5 V.

As shown in Figure 4d–f, the magnitude of R, P, and S/N reduces for shorter pulse widths, because of the finite slew rate of I_ds_. As an example, at V_gs_ = −7 V, by varying the light pulse width in the range from 100 ms to 1 s, R changes by a factor 30, while from 1 s to 10 s, R changes only by a factor ~2. A similar behavior is observed for P and S/N. As a consequence, the device characterizations achieved in quasi-stationary regimes are not representative of the device response to short light pulses, as expected in various photodetection applications.

**Figure 5:**
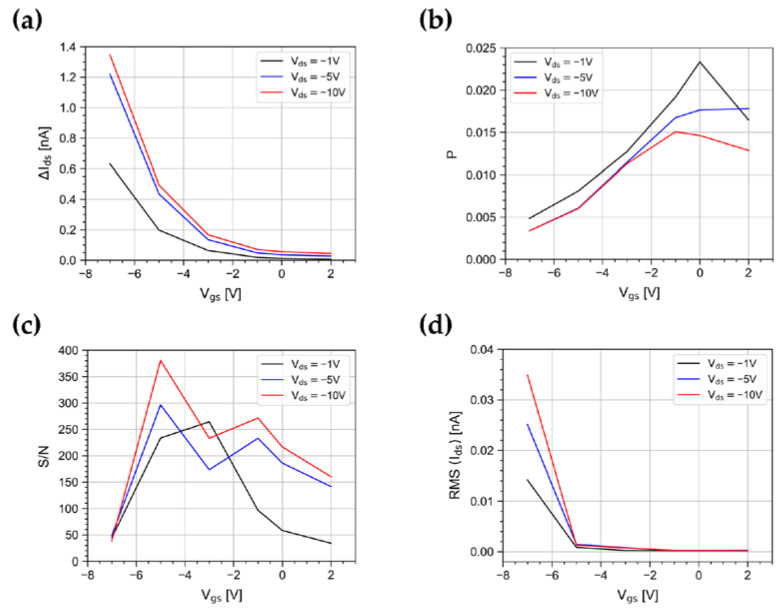
(**a**) Responsivity, (**b**) photosensitivity, (**c**) signal-to-noise ratio, and (**d**) root mean square of the I_ds_ dark current as a function of V_gs_ for V_ds_ = −1, −5, −10 V at an irradiance of 500 nW/cm^2^.

We studied R, P and S/N for two more V_ds_ values (−5 V, −10 V) and compared the results, as shown in Figure 5a–c. The responsivity monotonically increases with the absolute values of gate and drain voltages. From the measured values of R, we derived the external quantum efficiency (EQE) of the device, i.e., the ratio between the number of incident photons and photogenerated carriers, as follows [1]:

(3) $$\mathrm{EQE}=R\frac{hc}{\lambda q},$$

where *q* is the elementary charge, *h* is the Planck constant, and *c* is the speed of light in the vacuum. Values as high as 20% were measured at V_gs_ = −7 V and V_ds_ = −10 V. 

It should be noted that as V_gs_ and V_ds_ increase, the dark current I_ds_ grows as well, as shown in transfer curves in Figure 2b. As a consequence, in applications where the OPT is DC coupled to the front-end circuit, and the read-out of short light pulses asks for high-gain trans-impedance amplifiers, the increment of the dark current could bring the amplifier in saturation.

**Figure 6:**
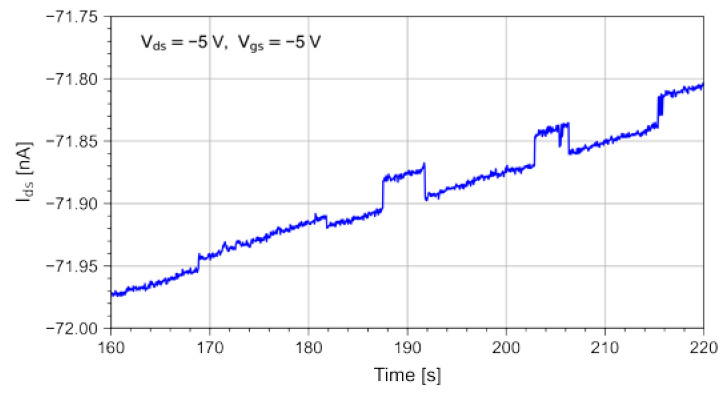
Random telegraph signal behavior in the drain dark current observed for the OPT polarization of V_ds_ = −5 V, V_gs_ = −5 V.

Differently from R, both P and S/N correlate the photoresponse with the dark current and its root mean square (Figure 5d). The highest photosensitivity value was obtained with the lowest drain bias applied in our tests (V_ds_ = −1 V) and with a gate voltage V_gs_ = 0 V. On the contrary, the S/N plot suggests the best operating point to be at V_gs_ = −5 V and V_ds_ = −10 V. However, for high values of V_ds_, it is well known that the high electrical field lowers the device stability [18]. Indeed, we have observed that random telegraph signal (RTS) phenomena arise. RTS consists of the discrete, fast fluctuation of the dark current I_ds_ between two or more values [38]. Figure 6 shows, as an example, a time window where the drain current was affected by RTS behaviors. The onset of RTS is clearly to avoid. The dark current fluctuations generate swift P variations and the step changes in the signal baseline could be interpreted as a photoresponse. A good compromise is then achieved by decreasing the gate and drain bias down to values as low as V_ds_ = −1 V and V_gs_ = −3 V. Taking all that into account, the optimal operating point clearly depends on the specific application and the experimental setup to be used.

**Figure 7:**
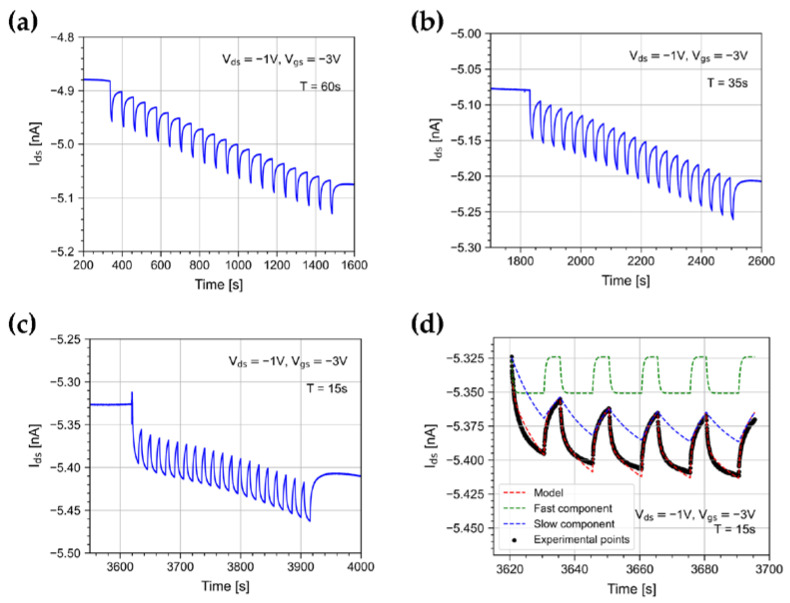
Drain current when illuminating the device with 10 s width light pulses with a period of (**a**) T = 60 s, (**b**) T = 35 s, and (**c**) T = 15 s. The OPT was polarized with V_ds_ = −1 V and V_gs_ = −3 V, and the irradiance was 500 nW/cm^2^. (**d**) Drain current measured under 10 s light pulses with a period of 15 s (black points) and the eq. 6 model best-fit curve (red dotted line). The fitted slow and fast model components are shown in green and blue, respectively.

In order to study possible channel stress effects due to light exposure, we illuminated the DUT with bursts of 20 pulses for different repetition periods: T = 15, 20, 35, 60 s. Figure 7 shows the drain current trend when illuminating the device with bursts of light pulses with different periods. As noted in [29], the exposure to repeated light pulses causes a drift of the drain current. This can be explained by the pile-up of a persistent component of the photocurrent [33,39,40].

Moreover, a pulse height reduction between the first pulse of the burst and the following ones is observed. A similar effect was shown in [41]; however, it has neither been discussed nor interpreted. Figure 7a–c clearly shows that the shorter the period between pulses, the greater the pulse height reduction.

We found that such phenomena can be described by composing models taken from the literature [12,42,43]. As suggested in [42,43], we assumed that the photocurrent I_’o_ is linearly dependent on the total number of photogenerated minority carrier traps n:

(4) $$I_{\mathrm{ph}}\left(t\right)\propto n\left(t\right)$$

The time evolution of the defect density is governed by a rate equation:

(5) $$\frac{dn}{dt}=a-bn\left(t\right),$$

where *a* is the defect generation rate, which is ultimately related to irradiance, and *b* is the defect recombination rate. 

The phenomena we observed require the presence of two populations of defects with densities *n_x_* and *n_y_*, whose activation energies are different [12]. In such an approach, *n_x_* is responsible for the fast photocurrent component, while *n_y_* for the persistent photocurrent component. Differently from [12], where two defect populations with continuous activation energy values were considered, here we simply assumed two discrete values. The overall photocurrent is hence given by:

(6) $$I_{\mathrm{ph}}\left(t\right)\propto n_{x}\left(t\right)+n_{y}\left(t\right)$$

where both *n_x_* and *n_y_* follow a rate equation in the form of Equation (5).

Figure 7d shows the measured drain current (in black) and superimposed the Equation (6) model best-fit curve (red dotted curve). The fast and persistent photocurrent components due to the *n_x_* and *n_y_* defect densities’ evolution are shown in green and blue, respectively. 

The model reproduces the decrease of the signal height observed in data between the first pulse and the following ones well. This phenomenon happens because of the build-up of the persistent photocurrent component, which after the first burst does not return to zero. The wander of the drain current baseline changes the response to the light pulses in the burst as a function of the burst length. 

**Figure 8:**
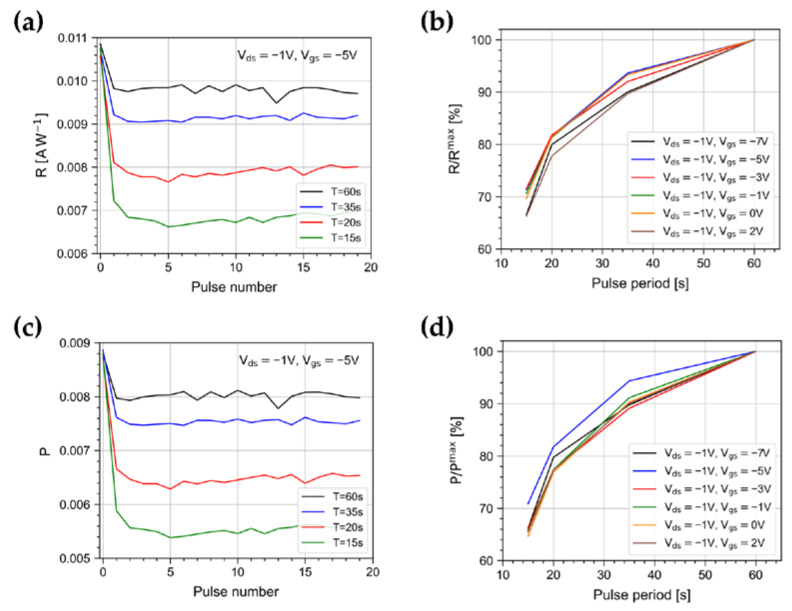
Trends of (**a**) R and (**c**) P for pulses in the burst, for different periods, at V_ds_ = −1 V and V_gs_ = −5 V. Averages of (**b**) R and (**d**) P in the burst, normalized to the values at 60 s, as a function of the light pulse period for different gate polarizations.

Figure 8a,c show the trend of R and P for the pulses of the burst for different periods. R and P show a marked reduction between the first and the second pulse, while they remain nearly constant afterward. Figure 8b,d show R and P values averaged over the burst pulses and normalized to the value achieved at 60 s, as a function of the light pulse period. They clearly show that the shorter the pulse period, the smaller are R and P. A similar trend is observed at different gate polarizations.

**Figure 9:**
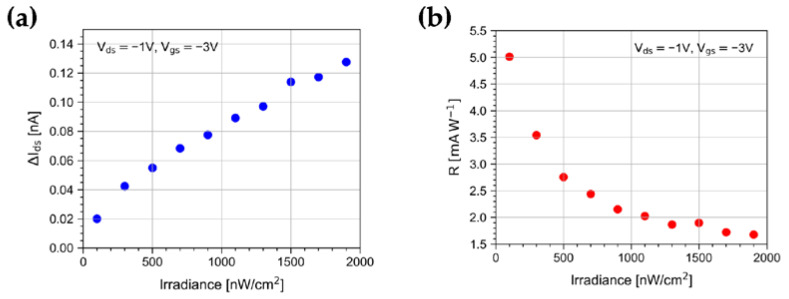
Trend of (**a**) ΔI_ds_ and (**b**) R for 10 s light pulses as a function of the irradiance at V_gs_ = −3 V and V_ds_ = −1 V.

Finally, we have investigated the OPT photoresponse to 10 s light pulses as a function of the irradiance in the range from 100 nW/cm^2^ to 1900 nW/cm^2^. As shown in Figure 9, ΔI_ds_ increases with the irradiance and values as high as ~120 pA are reached at 1900 nW/cm^2^. On the other hand, light pulses at an irradiance as low as 100 nW/cm^2^ still produce a detectable response of ~20 pA. A nonlinear behavior is observed in Figure 9a, which is likely to be attributed to the limiting nature of charge transport mechanisms in the polymer [44]. As a consequence, at the largest irradiance applied in our test, R reduces, and values as low as 1.5 mAW^−1^ are reached (Figure 9b).

## 4. Conclusions

Our results aim to evaluate the dynamic performance of a DNTT-based OPT in the view of deployment in applications that foresee the detections of fast, random light pulse sequences, such as radiation detection, dosimetry, and visible light communication. We have characterized the dynamic photoresponse to ~470 nm light pulses, close to the DNTT absorption peak. 

We investigated several FoMs, such as photosensitivity, responsivity, and signal-to-noise ratio, under various timing conditions, at different irradiances and operating points. In all the explored regions, we observed that photosensitivity, responsivity, and signal-to-noise ratio strongly depend on the timing characteristics of the light pulses. R changes by more than an order of magnitude from 100 ms to 1 s, while R changes only by a factor ~2 from 1 s to 10 s. Stationary conditions are reached only after an exposition of a couple of minutes. P and S/N behave in a similar fashion.

The maximum of the responsivity is obtained at the higher gate and drain voltages explored. Differently, the highest photosensitivity value is obtained with the lowest drain bias applied in our tests and with the gate and source shorted to ground. The S/N plots suggest increasing both V_gs_ and V_ds_ to achieve the best operating point. It is noticeable, however, that for high values of V_ds_, random telegraph signal effects in the drain current arise. Their occurrence makes it questionable to quantify the S/N ratio, and moreover, is clearly to be avoided, because such step-like fluctuations could be interpreted as a true signal. In applications where signal amplitude is paramount, both V_gs_ and V_ds_ should be increased to the limit allowed by the stability of the device operation and bias stress effects. On the other hand, in order to avoid saturation in DC-coupled high-gain trans-impedance amplifiers, a low dark current is mandatory, and hence low bias voltages are required.

We also studied stress and distortion effects in the photoresponse when pulse bursts are applied. We observed a reduction in the ΔI_ds_ photoresponse, between the first and the second pulse, while it remains approximately constant in the following pulses. We found that such an effect in the data is well reproduced by composing models taken from the literature, in which the photocurrent is assumed to be proportional to the defect density. Supposing the presence of just two kinds of defects with discrete activation energies allowed us to reproduce the experimental data accurately in a time window of minutes. 

In future works, we aim to correlate the OPT detectivity and the limit of detection to the timing of the incoming light pulses.

## Data Availability

The data presented in this study are available on request from the corresponding author.
